# Supplementary material for: Protein structural features predict responsiveness to pharmacological chaperone treatment for three lysosomal storage disorders
Source: PLoS Comput Biol. 2021 Sep 16;17(9):e1009370. doi: 10.1371/journal.pcbi.1009370 (PMC8478239; doi:10.1371/journal.pcbi.1009370)
Supplement: S1 Fig — (PDF) [file pcbi.1009370.s001.pdf]

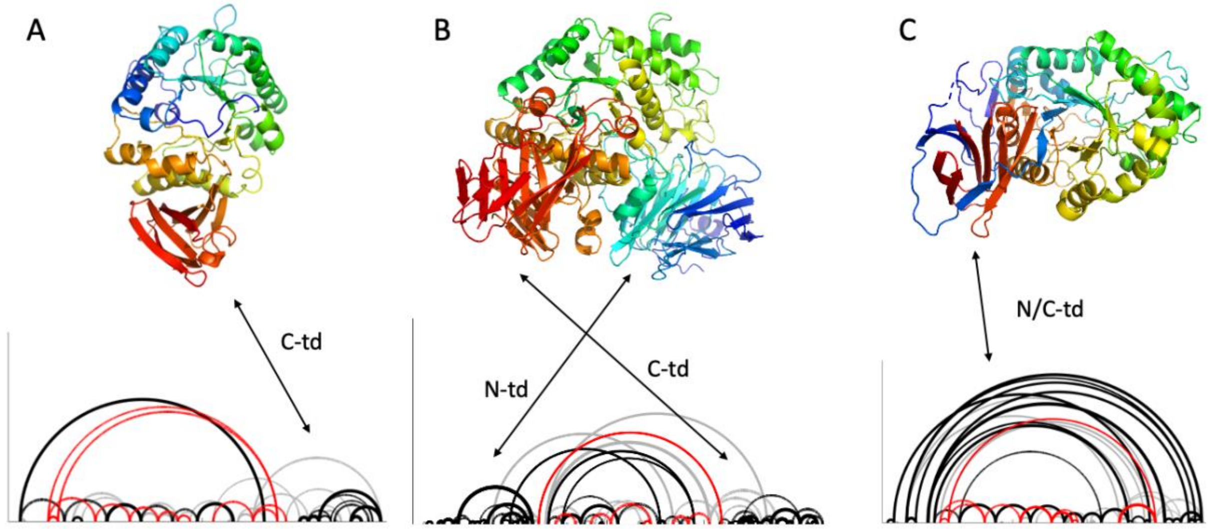


**S1 Fig. Diagrams of proteins with mutants associated with lysosomal storage disorders.** (A) alpha-galactosidase A (Fabry disease), (B) acid alpha-glucosidase (Pompe disease) (C) glucocerebrosidase (Gaucher disease). Topology diagrams are shown below the protein representations, where curves connect contacting secondary structural elements along the chain, with contacts between beta strands in black, contacts between alpha helices in. red, and other contacts in gray.
