# Supplementary material for: Protein structural features predict responsiveness to pharmacological chaperone treatment for three lysosomal storage disorders
Source: PLoS Comput Biol. 2021 Sep 16;17(9):e1009370. doi: 10.1371/journal.pcbi.1009370 (PMC8478239; doi:10.1371/journal.pcbi.1009370)
Supplement: S2 Fig — (PDF) [file pcbi.1009370.s002.pdf]

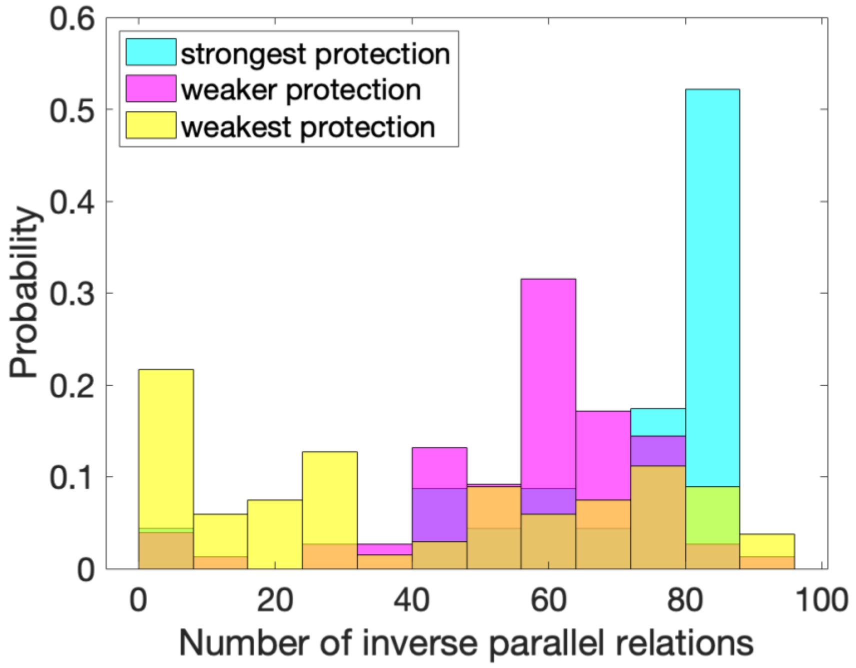


**S2 Fig. Number of inverse parallel relations, by protection from exchange in mass spectrometry experiments.** Crystal structure 1J5T is referenced. Strongest protection refers to residues in β4α4. Weaker protection is conferred in α1β2 and β5α5β6α6β7. Weakest protection refers to all other residues. T-test p values are: 0.005 for strongest and weaker protection, $6x10^{-5}$ for strongest and weakest protection, and $2x10^{-4}$ for weaker and weakest protection.
