# Supplementary material for: Protein structural features predict responsiveness to pharmacological chaperone treatment for three lysosomal storage disorders
Source: PLoS Comput Biol. 2021 Sep 16;17(9):e1009370. doi: 10.1371/journal.pcbi.1009370 (PMC8478239; doi:10.1371/journal.pcbi.1009370)
Supplement: S3 Fig — (PDF) [file pcbi.1009370.s003.pdf]

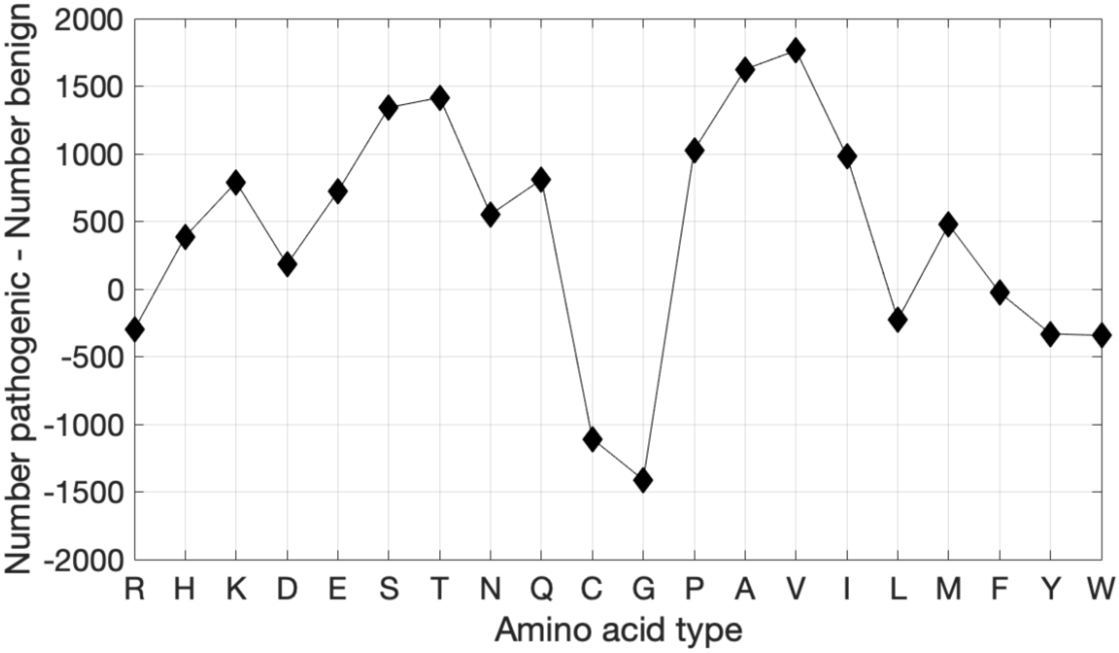

S3 Fig. Propensity for benign mutation by amino acid type. Shown is the number of benign mutations minus the number of pathogenic mutations, starting from the given residue, in the ADDRESS database (*1*) (including mutations that could not be mapped to a structure).
