# Supplementary material for: Protein structural features predict responsiveness to pharmacological chaperone treatment for three lysosomal storage disorders
Source: PLoS Comput Biol. 2021 Sep 16;17(9):e1009370. doi: 10.1371/journal.pcbi.1009370 (PMC8478239; doi:10.1371/journal.pcbi.1009370)
Supplement: S1 Table — (PDF) [file pcbi.1009370.s004.pdf]

S1 Table. Optimization of Fabry decision tree complexity

complexity parameter	0.01	0.012	0.014	0.016	**0.018**	0.02	0.022	0.024	0.026
MCC Pompe	0.412	0.443	0.443	0.443	**0.492**	0.492	0.492	0.492	0.36
MCC Fabry	0.513	0.449	0.449	0.449	**0.393**	0.376	0.376	0.376	0.323
Nodes	14	8	8	8	**5**	4	4	4	2
