# Supplementary material for: Protein structural features predict responsiveness to pharmacological chaperone treatment for three lysosomal storage disorders
Source: PLoS Comput Biol. 2021 Sep 16;17(9):e1009370. doi: 10.1371/journal.pcbi.1009370 (PMC8478239; doi:10.1371/journal.pcbi.1009370)
Supplement: S2 Table — (PDF) [file pcbi.1009370.s005.pdf]

S2 Table. Leading auto-ML models

Dataset	Best performing model
Fabry	StackedEnsemble_BestOfFamily_AutoML_20210422_151045
Pompe	XGBoost_grid__1_AutoML_20210422_152715_model_28
