# Supplementary material for: Protein structural features predict responsiveness to pharmacological chaperone treatment for three lysosomal storage disorders
Source: PLoS Comput Biol. 2021 Sep 16;17(9):e1009370. doi: 10.1371/journal.pcbi.1009370 (PMC8478239; doi:10.1371/journal.pcbi.1009370)
Supplement: Attachment — Submitted filename: Response_to_reviewers.pdf [file pcbi.1009370.s007.pdf]

Response to Reviewer #1

We appreciate the comments and suggestions from the Reviewer, which we found helpful in improving the quality of the manuscript. We included further references, as suggested, and we commented on how our work may relate to findings based on molecular dynamics simulations.

1. **<u>The Reviewer commented:</u>**

> ***General***
> ***The paper: Protein structural features predict responsiveness to pharmacological chaperone treatment for three lysosomal storage disorders by Jaie Woodard et al deserves to be published but needs changes and additions.***
> ***I found it very interesting the fact that the authors show the predictions for Gaucher disease generated using the Fabry disease or Pompe disease model separately.***

We thank the reviewer for positive comments and for suggestions on how to improve the paper. We added the following sentence at Page 5:

> and that predictions may be transferrable between proteins that cause differing lysosomal storage disorders.

2. **<u>The Reviewer commented:</u>**

> ***The only pharmacological chaperone that has been approved for therapy is galafold (trade name). The use of pharmacological chaperones for Fabry disease has been more successful and therefore a large number of papers and data have accumulated. The authors claim to have used data from Benjamin et al (BENJAMIN, Elfrida R., et al. The validation of pharmacogenetics for the identification of Fabry patients to be treated with migalastat. Genetics in Medicine, 2017, 19.4: 430-438), but other papers also report data on many Fabry mutations and should be cited because they report exhaustive numerical data from independent groups (CITRO, Valentina, et al. The large phenotypic spectrum of Fabry disease requires graduated diagnosis and personalized therapy: A meta-analysis can help to differentiate missense mutations. International journal of molecular sciences, 2016, 17.12: 2010). It is therefore no wonder that the model derived from the data for alpha-galactosidase is more robust than that derived for alpha-glucosidase as observed by the authors themselves in the discussion (In general, based on our statistics and properties of the training data and models, we might expect the machine learning (H2O) results based on the Fabry dataset to yield the highest accuracy results) and table I . I think this point should be stressed further.***

We included the recommended reference (Ref. 13) and other references relevant to lysosomal storage disorders (Ref. 16). We added a sentence stressing the accuracy of the referenced machine learning method (Page 10):

> The larger dataset of Fabry disease, combined with the sophisticated machine learning method, yields increased performance over the other methods described.

3. **<u>The Reviewer commented:</u>**

Other works have tried to predict responsive mutations for Fabry disease. In particular one paper (CUBELLIS, Maria Vittoria; BAADEN, Marc; ANDREOTTI, Giuseppina. Taming molecular flexibility to tackle rare diseases. Biochimie, 2015, 113: 54-58.)demonstrates that mutations occurring in the most flexible sites are generally more responsive. but there are exceptions in particular for glycines. It would be interesting to understand if the method used by the authors allows to explain why mutations in flexible glycines are less responsive than expected and in general the accordance of their results with those obtained using molecular dynamics.

We thank the reviewer for drawing our attention to this paper, which we cited as Ref. 29. We referenced results from our ADDRESS database (Ref. 3) and included a new figure (S3 Fig), as well as referencing work by the Skolnick group (Ref. 48), which shows that mutations from glycine to other residues are in general more likely to be pathogenic vs. benign. This, possibly in addition to the intrinsic flexibility of glycine residues, may account for the molecular dynamics result noted. We added the following paragraph to discuss the point at Page 11:

A molecular dynamics study (29) reported that glycine residues in particular had lower residual activity than might be expected based on RMSF, in relation to other residues. Glycine in general may be expected to have high flexibility, due to the large amount of torsional accessibility in the Ramachandran map relative to other residues; in addition, referencing our work summarizing mutations in 70,597 proteins (3), and as also seen previously (48), mutations from glycine to other residues tend to be pathogenic more often than mutations from most other residues (see S3 Fig), which could be a reason for the observed anomalous behavior of glycine mutations.

4. <u>The Reviewer commented:</u>

The originality of the work consists in trying to use a model protein for which there are many in vitro experimental data for the prediction of the effect of chaperones on other less studied proteins. I would advise the authors to bolster their data by verifying what would happen if the alpha-glucosidase (Pompe disease) data were used to "predict" the data for alpha galactosidase (Fabry disease) and vice versa.

Table 3 contains information on the MCC for Fabry disease data predicting Pompe disease data and for Pompe disease data predicting Fabry disease data. The MCC in the former case is much higher than in the latter case, possibly because the Fabry disease dataset contains substantially more data. We added the following sentence to clarify this approach (Page 10):

In addition to cross validation on the same dataset, we obtained MCC using the other dataset as the test set. (i.e., training on the Fabry dataset and testing non the Pompe dataset, or training on the Pompe dataset and testing on the Fabry dataset).

5. <u>The Reviewer commented:</u>

Moreover
In the Introduction
"… Pharmacological chaperones have become popular as potential treatments for several diseases, including lysosomal storage disorders, "
More correctly "some" lysosomal storage disorders

The same applies in the discussion:
"Pharmacological chaperone treatment has been explored in the case of several diseases (9, 10, 16), 317 with much of the focus centering on lysosomal storage disorders"

The trademark of the pharmacological chaperone for Fabry disease is GALAFOLD™ (migalastat): the authors should mention this

We thank the reviewer for pointing out the issues. Corrections have been made (see Pages 4, 11, and 4, respectively).

6. <u>The Reviewer commented:</u>

"The drug binds the protein as an inhibitor (although non-inhibitory chaperones have also been proposed, which may overcome dosing complications introduced by inhibitory effects), assisting folding in the Endoplasmic Reticulum."

References should be provided at least for the three lysosomal storage diseases that are analysed by the authors

Relevant and additional references are provided in Ref. 9, 14-16, 18-20.

7. <u>The Reviewer commented:</u>

In the Discussion

The authors make a generic comment concerning Arg to Cys mutations.

Indeed these variants have been reviewed for Fabry disease in (CITRO, Valentina, et al. The large phenotypic spectrum of Fabry disease requires graduated diagnosis and personalized therapy: A meta-analysis can help to differentiate missense mutations. International journal of molecular sciences, 2016, 17.12: 2010.)and the authors can find data in the supplementary files. R118C, R363C, R49C show different values of residual basal activity in the absence of the chaperones but are all responsive in particular R363C.

We made note of this in the Discussion (Page 12):

In fact, in the case of Fabry disease, several arginine to cysteine mutations show residual basal activity and responsiveness to pharmacological chaperone treatment (13).

Response to Reviewer #2

We thank the reviewer for helpful comments. We responded to important details regarding the druggability of lysosomal storage disorders.

1. **The Reviewer commented:**

In this manuscript Woodard et al perform a structural analysis of variants within three inherited lysosomal storage disorders, comparing variants responsive to pharmacological chaperone treatment to those unresponsive to such treatment.
The article is interesting and relevant for the field of genetic diseases and precision medicine and drug discovery.

We appreciate the positive comments from the Reviewer on the work.

2. **The Reviewer commented:**

Comments:
1. Please, discuss if the the stabilization of mutant proteins can also be achieved by the substrate of the enzyme, cofactors or coenzymes.

We found that, historically, the product galactose played a role in drug discovery, although it was not itself feasible to use as a treatment. Accordingly, we added the following discussion to the Introduction section (Page 4):

This follows an initial observation that the product and inhibitor galactose, of which Migalastat is an analog, improves activity and patient condition, although it is not a feasible therapeutic due to the high required dose (*9*, *17*).

3. **The Reviewer commented:**

2. In Gaucher disease, the L444P variant can be rescued by a L-idonojirimycin derivative, PMID: 28171725 and PMID: 2604518

We thank the reviewer for insightful comments. We added a note to Table 1:

*Although non-responsive to N-nonyl-deoxynojirimycin, L444P was shown to be responsive in other cases (43, 44).
